# De novo mutations in *ARID1B* associated with both syndromic and non-syndromic short stature

**DOI:** 10.1186/s12864-015-1898-1

**Published:** 2015-09-16

**Authors:** Yongguo Yu, RuEn Yao, Lili Wang, Yanjie Fan, Xiaodong Huang, Joel Hirschhorn, Andrew Dauber, Yiping Shen

**Affiliations:** Department of Pediatric Endocrinology/Genetics, Xinhua Hospital, Shanghai Jiao Tong University School of Medicine, Shanghai Institute for Pediatric Research, 1665, Kongjiang Road, Shanghai, 200092 China; Medical Genetics Department, Shanghai Children’s Medical Center, Shanghai Jiaotong University School of Medicine, 1678 Dongfang Road, Shanghai, 200127 China; Division of Endocrinology and Genetic Metabolism, Department of Internal Medicine, Shanghai Children’s Medical Center, Shanghai Jiaotong University School of Medicine, 1678 Dongfang Road, Shanghai, 200127 China; Division of Endocrinology and Division of Genetics, Children’s Hospital Boston, 300 Longwood Ave, Boston, MA 02115 USA; Program in Medical and Population Genetics, Metabolism Program, Broad Institute, Cambridge, MA 02141 USA; Center for Basic and Translational Obesity Research, Children’s Hospital Boston, Boston, MA 02115 USA; Division of Endocrinology, Cincinnati Children’s Hospital Medical Center, Cincinnati, OH 45229 USA; Department of Laboratory Medicine, Boston Children’s Hospital, Boston, MA USA; Department of Pathology, Harvard Medical School, Boston, MA 02115 USA; Claritas Genomics, Cambridge, MA 02139 USA

**Keywords:** *ARID1B*, Short stature, Haploinsufficiency, Coffin-Siris Syndrome, Chromatin remodeling

## Abstract

**Background:**

Human height is a complex trait with a strong genetic basis. Recently, a significant association between rare copy number variations (CNVs) and short stature has been identified, and candidate genes in these rare CNVs are being explored. This study aims to evaluate the association between mutations in *ARID1B* gene and short stature, both the syndromic and non-syndromic form.

**Results:**

Based on a case-control study of whole genome chromosome microarray analysis (CMA), three overlapping CNVs were identified in patients with developmental disorders who exhibited short stature. *ARID1B*, a causal gene for Coffin Siris syndrome, is the only gene encompassed by all three CNVs. A following retrospective genotype-phenotype analysis based on a literature review confirmed that short stature is a frequent feature in those Coffin-Siris syndrome patients with *ARID1B* mutations. Mutation screening of *ARID1B* coding regions was further conducted in a cohort of 48 non-syndromic short stature patients,andfour novel missense variants including two *de novo* mutations were found.

**Conclusion:**

These results suggest that haploinsufficient mutations of *ARID1B* are associated with syndromic short stature including Coffin-Siris syndrome and intellectual disability, while rare missense variants in *ARID1B* are associated with non-syndromic short stature. This study supports the notion that mutations in genes related to syndromic short stature may exert milder effect and contribute to short stature in the general population.

**Electronic supplementary material:**

The online version of this article (doi:10.1186/s12864-015-1898-1) contains supplementary material, which is available to authorized users.

## Background

Human height is a quantitative trait that follows a Gaussian distribution. Short stature is typically defined as a height more than 2 standard deviations (SD) below the corresponding mean height for a given age, gender and ethnic population. Individuals with short stature include those at the tail of the normal distribution (not necessary associated with any disorders) as well as individuals with rare disorders that restrict growth. Genome-wide association studies (GWAS) have identified over 400 independent loci associated with height in the general population which collectively explain ~20 % of the variation in adult height [1]. Rare variants with larger effects have been found in a number of genes leading to syndromic short stature disorders. For example, the *SHOX* gene was identified as the gene responsible for short stature in Turner syndrome [2] and Leri-Weill syndrome [3]. Recently, we demonstrated a significant association between low-frequent copy number deletions and short stature, supporting the hypothesis that rare haploinsufficient genes play significant roles in human growth [4]. We further demonstrated that an increased burden of rare deletions may also contribute to short stature in a non-clinically ascertained population, underscoring the concept that milder defects in genes known to cause syndromic short stature may contribute to short stature in the general population.

In this study, we focus on a recurrent CNV detected in patients with short stature. We propose that the *ARID1B* gene, which is the only gene intercepted by three CNVs, is a novel short stature gene. We provide additional evidence supporting that *ARID1B* mutations are associated with both syndromic and non-syndromic short stature.

## Methods

### CNV detection and evaluation

Whole genome microarray (Agilent 244K) was performed to detect structural variants in a clinical cohort. Subjects were eligible if they had a height measurement recorded between the ages of 2 and 20 years and had a chromosomal microarray performed as part of their clinical evaluation. All information was obtained with appropriate consent from Boston Children’s hospital [M09-06-0290]. Subjects with aneuploidy and poor microarray quality were not included, leaving a final sample size of 4,411 individuals including 415 patients with short stature, 196 patients with tall stature and 3800 patients with normal stature. All CNV data were called with NEXUS software (BioDiscovery, El Segundo, California). The recurrent copy-number variations involving *ARID1B* gene were validated by multiplex ligation-dependent probe amplification (MLPA). MLPA probe and reagents from MRC Holland (SALSA MLPA P433 ARID1A-ARID1B probemix). Data analysis and visualization was done on Coffalyzer software. A CNV is defined as non-benign when it does not overlap with CNV reported in DGV (Database of Genomic variants) or overlaps with CNV with less than 1 % population frequency in DGV. Non-benign recurrent or overlapping CNVs were identified in the subjects with short stature and compared to their occurrence in normal stature population. The UCSC genome browser’s custom track was used to depict the overlapping nature of CNVs and to delineate the minimal region of overlap (MRO).

### Literature review of height of patients with *ARID1B* deletion or mutation

We identified a total of 70 individuals carrying *ARID1B* deletions or mutations from a Pubmed search and the DECIPHER database. 65 of them had information on height. We converted all height parameters available to Z-scores based on CDC growth charts (http://www.cdc.gov/growthcharts/zscore.htm).

### Mutation screening in 48 non-syndromic short stature Chinese patients

Forty-eight non-syndromic short stature Chinese patients were recruited in Shanghai Children’s Medical Center. Their age, gender and height information are included in Additional file 1. The inclusion criteria were individuals with height below 3rd percentile without a clinical diagnosis of intellectual disability or developmental delay. All information was obtained with appropriate consent based on requirements of Shanghai Children’s Medical Center【SCMC-IRB-K2013007】.Subjects were randomly selected in non-syndromic short stature patients. Since it is unclear yet if *ARID1B* affects hormone-related pathways, we did not use hormonal status as a criteria for subject selection. Genomic DNA was extracted from peripheral blood of all participants using QIAamp Blood DNA Mini kit®. Mutation screening for all coding regions of *ARID1B* were done by Polymerase chain reaction (PCR) amplification followed by Sanger sequencing. Sequence variants were evaluated with mutation surveyor (Soft Genetics, State College, PA) and their potential functional impact was predicted using *insilico* prediction programs including SIFT [5], Polyphen2 [6], Condel [7] and Align-GVGD [8]. Paternity tests were performed with short tandem repeat (STR) markers for the two probands with de novo variants (using AmpFLSTR® Identifiler® PCR amplification kit). The study was reviewed and approved by the SCMC ethical committee and all participants or their parents signed an informed consent form. In addition, we compared the variant frequency in the *ARID1B* coding regions detected by exome sequencing to that of 494 normal Chinese controls. The normal Chinese controls were age and gender matching Chinese individuals of normal height, weight and were recruited from multiple geographic areas for an effort to create a common sequence variants database of normal Chinese children.

## Results

### Copy number imbalances involving *ARID1B*in patients with short stature and developmental disorder

**Fig. 1:**
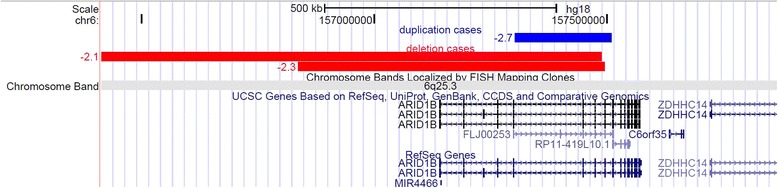
Three individuals with copy number variants encompassing *ARID1B* shown as tracks in the UCSC genome browser

Four thousand four hundred eleven individuals in the clinical population met our inclusion criteria (as described in methods), including 415 patients with short stature, 196 patients with tall stature and 3800 patients with normal stature. Three individuals with copy number variants encompassing *ARID1B* were identified among 415 patients with short stature who underwent clinical microarray analysis at Boston Children’s Hospital. The locations of the two deletions and one duplication in relation to the *ARID1B* gene are shown in Fig. 1.

Figure 1

Red tracks indicate deletion and blue track indicates duplication. The numbers associated with each track are the Z-scores of patient’s height.

The first patient (patient A) carries a 658 kb *de novo* deletion at 6q25.3 which affected only *ARID1B*; the second patient (patient B) carries a 6.8 Mb de novo deletion at 6q25.1-q25.3 which overlaps with the deletion in Patient 1. The height Z-score for both patients are -2.3 and -2.1 respectively. The patient with the duplication (patient C) was a 13-year-old Caucasian girl who carries a 207 Kb maternally inherited intragenic duplication at 6q25.3. Her height Z-score was -2.7. The mother’s height is below average (Z = -0.33) but not short. In addition to short stature, all three patients exhibited language impairment, facial malformation and intellectual disability. Thus all three patients exhibited syndromic short stature. Detailed clinical phenotypes and their growth curves can be found in Additional files 1 & 2.

There were no CNVs involving *ARID1B* in 3800 patients with normal stature and 196 patients with tall stature in our clinical population.

### Short stature is a frequent feature associated with patients with *ARID1B* mutations

A total of 70 patients with mutations in *ARID1B* (including translocation, deletion, duplication, nonsense and truncating mutations) have been described in the literature and Decipher database, many of whom have Coffin-Siris syndrome. We summarize the available clinical features of these patients in Table 1. Five patients had no height information. 22 out of the remaining 65 patients (33.8 %) had short stature. 90 % of patients have height Z-scores below -1 SD. None of the patients had height Z-scores above 0 SD. The average available Z score among patients with mutations in *ARID1B* is -1.86 SD. Thus, growth retardation and short stature is a common feature associated with mutations in *ARID1B* and Coffin-Siris syndrome.

**Table 1:** Clinical features of patients with *ARID1B* mutations

Cases from literature review																										
Case ID^a^	1	2	3	4	5	6	7	8	9	10	11	12	13	14	15	16	17	18~45		46	47	48	49	50	51	52
Gender	M	F	M	F	F	F	F	F	F	F	M	M	F	F	F	M	M	NS		F	M	F	M	M	F	F
Genomic defect^b^	Trans	Del	Del	Del	Del	Del	Del	Del	Del	Dup	Nonsense	Fx	Nonsense	Fx	Fx	Nonsense	Fx			Del	Del	Del	Del	Del	Del	Del
Height (age)	117 cm (7.5 y)	84 cm (2 y 11 m)	1.59 cm (46 y)	44.5 cm (at birth)	66.8 cm (9 M)	152 cm (18 y)	124 cm (8 y 9 m)	112 cm (9.5 y)	10th (3 y 3 m)	3–10th (4 y 11 m)	3-10th (3 y 5 m)	<3rd (7 y 3 m)	<3rd (12 y 8 m)	10–25th (4 y)	25–50th (6 y 3 m)	50th (17 y)	<3rd (20 y)	11 % (3/27) <−2.5SD		NS	NS	NS	52 cm (50th )	89.2 cm (2 y 9 m)	48 cm (at birth)	64 cm (10 m)
Z-score	−1.8	−2.5	−3.5	<−2.0	−2	−1.65	−2	−4	−1.2	−1.5	−1.5	−2	−2	−1.2	−0.6	0	−2			−1.5	−3.5	−2.6	0	−1.34	−1.65	−2.7
OFC (age)	+1.4 SD	−0.7 SD	−0.75 SD	NA	Z=0	25–50th	Z=0	−2.5 SD	10–25th	<3rd	50th	25–50th	25–50th	75th	75th	>97th	<3rd	0 % (0/27) <−2.5SD		NS	Z=−0.75	NS	75th	46.2 cm	31 cm	Z=−4.3
DD/ID	+	+	+	+	+	+	+	+	+	+	+	+	+	+	+	+	+	89 % (25/28)		+	+	+	+	+	+	+
ACC	+	NA	NA		NA	Partial	Partial	+	+	+	NA			NA	+	NA		36 % (9/25)		NS	NS	+	NS	NS		+
Hypotonia	+	+	NS	+	+	+	+	+	+	+	+	+	+	+	+			85 % (23/27)		NS	NS	NS	+	NS	+	NS
Seizure			NS			+	+	+					+		+	+		20 % (5/20)		NS	NS	NS	NS	NS	+	NS
ASD	+	+	+	+	NS	+					+							NS		NS	NS	NS	NS	NS	NS	NS
Language impairment	+	+	+	+	+	+	+	+	+	+	+	+	+	+	+	+	+	100 % (28/28)		+	+	+	+	NS	+	+
Malformation																										
Coarse facial	NS	NS	+	NS	NS	NS	NS	NS	NS	NS	NS	NS	NS	NS	NS	NS	NS	NS		+	+		NS	+	+	+
Low hair line	+	NS	NS	+	NS	+	+	+	NS	NS	NS	NS	NS	NS	NS	NS	NS	64 % (18/28)				+	NS	NS	NS	NS
Hypertrichosis		+	NS	NS	NS	NS	+	+						+	+			93 % (26/28)					NS	NS	NS	NS
Ear	+	NS	+	NS	NS	NS	NS	+	+	+	+	+	+	+	+	+	+	50 % (14/28)		NS	NS	NS	+	+	+	+
Eye	+	NS	+	+	+	+	+	+	+	+	+	+	+	+	NS	+	NS	65 % (17/26)		+	+	+	+	+	+	+
Nose	+	NS	+	+	NS	+	NS	+	+	+	NS	+	+	+	+	NS	NS	52 % (14/27)		NS	NS	NS	+	+	+	+
Mouth/lips/palate	+	+	+		+	+	+	+	NS	+	+	+	+	+	+	NS	+	78 % (21/27)		NS	NS	NS	+	+	+	+
Skeletal	NS	+	+	NS	NS	+	NS	+	NS	+	NS	NS	NS	NS	NS	NS	+	17 % (2/12)		NS	NS	NS	NS	NS	NS	NS
Limb/extremeties	+	+	+	+	+	+	+	+	NS	+	+	+	+	+	NS	NS	+	>75 %		+	NS	NS	+	NS	NS	+
	Cases from literature review													Cases from decipher database						Our cases from microarray and sequence						
Case ID	53	54	55	56	57	58	59	60	61	62	63	64		65	66	67	68	69	70	A	B	C	D	E	F	G
Gender	M	F	M	M	M	M	M	M	M	F	0	0		M	F	F	NS	NS	M	M	M	F	M	M	F	F
Genomic defect	Del	Del	Del	Del	Del	Del	Del	Fx	Nonsense	Nonsense	Fx	Del		Del	Del	Del	Del	Del	Dup	Del	Del	Dup	Missense	Missense	Missense	Missense
Height (age)	69.5 cm (10 m)	66 cm (9 m)	63.5 cm (7 m)	162 cm (16 y 6 m)	47 cm (14 days)	55.5 cm (at birth)	46.6 cm (3 m)		2/5 has short stature					Short stature	NS	NS	NS	NS	NS	<3rd	3rd	−3 SD (12 y 8 m)	−3 SD (3 y 6 m)	−3 SD (3 y 2 m)	−2 SD (9 y 8 m)	−2 SD (8 y 9 m)
Z-score	−1.56	−2.0	−2.0	−1.7	<−1.65	−1.65	<−3.0							−2	NS	NS	NS	NS	NS	−2.3	−2.1	−2.7	−2	−2	−2	−2
OFC (age)	Z=−4.5	Z=0	Z<−2.0	53 cm	Z<−1.65	Z=1.65	Z<−3.0	NS	NS	NS	NS	NS		NA			NS	NS	NA	50th	2nd	98th	NS	NS	NS	NS
DD/ID	+	+	+	+	+	+	+	+	+	+	+	+		+	+	+	+	+	+	+	+	+				
ACC	+	+	NS	NS	+	NS	+	NS	NS	NS	NS	NS		NA	+	NS	NS	NS	NS	+	+		NS	NS	NS	NS
Hypotonia	+	+	NS	NS	NS	+	+	4/5 have hypotonia						NA	+	NS	NS	NS	NS	+	+	+				
Seizure	NS	NS	NS	+	NS	NS	NS	2/5 have seizure						NA	NS	NS	NS	NS	+	+						
ASD	NS	NS	NS	+	NA	NA	NA	NS	NS	NS	NS	NS		+	NS	NS	NS	+	NS		+					
Language impairment	NS	NS	NS	+	NS	NS	NS	NS	NS	NS	NS	NS		+	NS	NS	NS	+	NS	+	+	+				
Malformation																										
Coarse facial	+	+	NS	NS	NS	+		+	+	+	+	+		+	NS	NS	NS	+	NS	+	+					
Low hair line	NS	NS	NS	NS	NS	NS	NS	NS	NS	NS	NS	NS		+	NS	NS	NS	NS	NS		+	+				
Hypertrichosis	NS	NS	NS	NS	+	NS	NS	+	+	+	+	+		NS	NS	NS	NS	+	NS		+					
Ear	+	+	NS	+	+	+	+	4/5 have abnormal ears						NS	NS	+	+	NS	NS	+	+	NS				
Eye	+	+	+	+	+	+	+	1/5 with vision problem						+	NS	+	+	NS	NS	+	+	+				
Nose	+	NS	+	+	+	+	+	+	+	+	+	+		+	NS	NS	NS	NS	NS	+	+	NS				
Mouth/lips/palate	+	+	+	+	NS	NS	+	+	+	+	+	+		+	NS	NS	NS	NS	+	+	+	+				
Skeletal	NS	NS	NS	NS	NS	NS	NS	3/4 have spinal anomalies						NS	NS	NS	NS	NS	+	+	+	+				
Limb/extremeties	NS	+	+	+	+	NS	+	+	+	+	+	+		+	NS	NS	+	NS	NS	+	+	+				

^a^1–8 Halgren et al., 9–17 Hoyer et al., 18–45 Santen et al. (2013), 46-48 santen et al. (2012), 49 Michelson et al., 50–53 Nagamani et al., 54 Pirola et al., 55 Narahara et al., 56–57 Sukumar et al., 58 Hopkin et al., 59 Meng et al., 60–64 Tsurusaki et al., 65–70 from decipher database

^b^The following abbreviations are used: *F* female, *M* male, *OFC* occipital-frontal circumference, *+* present, *−* absent, *NA* not analyzed, *NS* not stated, *ACC* agenesis of corpus callosum, *ASD* atrial septum defect, *trans* translocation, *del* deletion, *dup* duplication, *fx* frameshift

### *ARID1B* mutations in non-syndromic patients with short stature

**Fig. 2:**
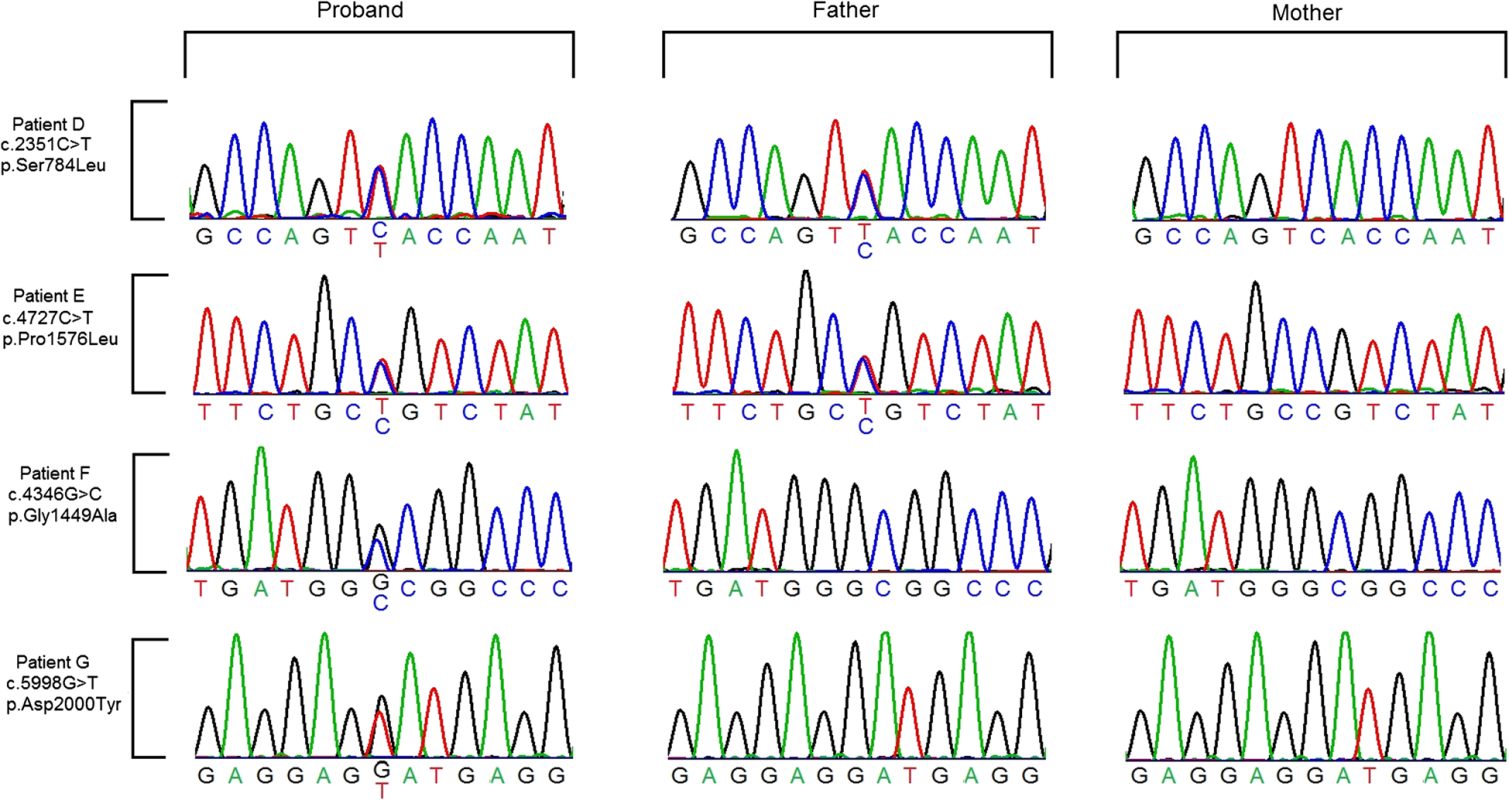
*ARID1B* Sanger sequencing data of four patients and their parents

We sequenced the coding regions and intron-exon boundaries of *ARID1B* in 48 non-syndromic short stature Chinese patients. We detected four missense variants (Fig. 2). Variants c.2351C > T and c.4727C > T in patients D and E respectively were inherited from their fathers and the c.2351 variant was present in a sister of normal height as well. Variants c.4346G > C and c.5998G > T in patients F and G were not identified in either parent. Paternity test confirmed the biological relationship between the probands and their parents (data available in Additional file 3), thus these two missense variants are *de novo* changes in the probands. Provocative growth hormone (GH) testing with intravenous infusion of Arginine and oral administration of clonidine was performed in the patients, following routine procedures. Patient E, F and G exhibited partial growth hormone deficiency by provocative GH testing (5-7ng/ml).Patient D had a normal growth hormone level (15.272ng/ml). None of them had intellectual disability or language impairment. Thus they are all considered to have non-syndromic short stature. Detailed clinical information of the four patients is presented in Additional file 1.

The four missense variants were all predicted to be deleterious by SIFT, PolyPhen2 and Condel (Additional file 4). They were novel variants and absent from 494 Chinese controls, the NHLBI Exome Variant Server 6500 dataset (http://evs.gs.washington.edu/EVS/) and the ExAC database (http://exac.broadinstitute.org/).

We detected 8 novel variants predicted to be deleterious in 494 Chinese controls. There is a significant enrichment of novel deleterious missense variants detected in short stature patients when compared to the normal Chinese controls (*p* = 0.016 Fisher exact test). It is unclear if the inherited variants in Patients D and E are pathogenic given the lack of segregation with short stature in the family. Partial penetrance of these two inherited variants are possible. Based on the recently published guideline for variant interpretation [9], the two *de novo* variants are classified as likely pathogenic and the other two variants are of uncertain significance (see Additional file 4).

## Discussion

High resolution array CGH enables the detection of small CNVs involving one gene or part of a gene. This approach allowed for the identification of candidate short stature genes. We scanned the whole genome for single CNVs in individual patients or overlapping CNVs with a single gene in the region of overlap in patients with short stature. We paid particular attention to *de novo* CNVs only present in patients and not in a control population. By this approach, we identified a chromatin-remodeling gene *ARID1B* as a novel short stature gene. *ARID1B* was the only gene in the region of overlap involving two deletions and one duplication. The two deletions were *de novo. ARID1B* has been identified as one of the causal genes for Coffin-Siris syndrome and it has also been associated with syndromic intellectual disability [10]. While growth retardation was mentioned as one of the features of Coffin-Sirissyndrome [11], this feature is currently under appreciated [12]. We then performed a comprehensive case review of the clinical features of all individuals carrying mutation in *ARID1B* regardless of their associated syndromes (Table 1). This genotype-driven approach revealed a significant association between short stature and mutations in *ARID1B*. Next, we further explored the possible contribution of *ARID1B* mutations to non-syndromic short stature by screening 48 short stature patients who did not have developmental delay or intellectual disability. As a result, we identified four novel missense variants in *ARID1B* including two *de novo* variants. The likelihood of a chance finding of two *de novo* variants in the same gene in 48 individuals is extremely small, indicating that these variants are likely causal of the patients’ short stature. Collectively, our data support the notion that *ARID1B* mutations cause growth retardation in syndromic patients and may also contribute to non-syndromic short stature.

### Coffin-Siris syndrome and short stature

Recently, loss of function mutations in *ARID1B* were identified as causative for Coffin-Siris syndrome, a rare genetic condition characterized by growth deficiency, developmental delay, severe speech impairment, intellectual disability, and specific physical features including microcephaly, coarse facial features, hypertrichosis, hypoplastic or absent fifth fingernails or toenails and hypoplasia or agenesis of the corpus callosum [13]. Our patient A exhibited many features of Coffin-Siris but was not noted to have hypoplastic or absent fifth fingernails or toenails. Our patient B showed typical signs of Coffin-Siris syndrome but not patient C. All three patients exhibited short stature. We performed a comprehensive review on published cases with loss-of-function mutations in *ARID1B* (Table 1). Based on the available clinical information from the published literature, all patients showed developmental delay (70/70) and language impairment (63/63). Most of patients exhibited hypertonia (49/56), seizures (16/52), agenesis of corpus callosum (23/47), autism or autistic features (9/42) as well as dysmorphic features: low-set or abnormal-shaped ears (42/47), prominent nose (41/55), various eye-related features (51/64), hypertrichosis (38/51), coarse face/abnormal head shape (16/18), low hair line (9/11), and spinal/skeletal anomalies (10/12). Patients ascertained with Coffin-Siris syndrome also exhibited dysplastic nails.

Case review data showed that 34 % of patients with *ARID1B* mutations had short stature defined as height below -2 SD. The majority (90 %) had a height Z-score below -1 and none of the patients had above average height. The average Z score among patients for whom height data was available is -1.86. Thus, growth retardation and short stature is a common feature associated with mutations in *ARID1B* and Coffin-Siris syndrome.

Coffin-Siris syndrome is a nucleosome remodeling complex (SWI/SNF-SWI) disorder. Mutations in other genes involved with the SWI/SNF complex such as *ARID1A*, *SMARCA2*, *SMARCA4*, *SMARCB1* and *SMARCE1* are also responsible for Coffin-Siris syndrome [12]. Recent genotype-phenotype analysis indicated that short stature is a prominent feature in patients carrying mutations in those other SWI/SNF genes [14] as well. Among all the Coffin-Siris syndrome patients detected with mutations in those BAF-complex-related-genes (BRG1/brm-associated factor), 21 % of them (9/43) showed short stature. Additionally, 24 % of Coffin-Siris patients without identified mutations presented with short stature. Thus short stature is a common feature of Coffin-Siris syndrome.

### *ARID1B* and non-syndromic short stature

We identified four novel missense variants among 48 individuals with idiopathic short stature not associated with developmental delay. These variants were not reported in 1000 genomes project database, dbSNP database, the ESP6500 dataset or ExAC database. Functional predictions suggest deleterious effect on protein for all four variants, although two of the variants were found in family members without short stature. All four variants were absent from 1100 ethnically matched controls. We found significantly fewer deleterious novel missense variants in age, gender, and ethnicity-matched controls than in short stature patients. Importantly, two of the variants were *de novo* changes not detected in parents and non-affected siblings. All four patients carrying *ARID1B* mutation showed no signs of Coffin-Siris syndrome, or developmental or mental deficits. *ARID1B de novo* mutations have been shown to contribute to the heritable complex traits, such as autism spectrum disorders (ASDs), although the effect size may be small [15]. We have not established the causal relationship between these mutations and short stature, but we postulate that *ARID1B* is also involved with idiopathic short stature in a similar manner.

Almost all 70 syndromic individuals carried loss-of-function *ARID1B* mutations which include genic deletions (*n* = 27), genic duplications (*n* = 2), nonsense mutations (*n* = 14), frame shift mutations (*n* = 21) and a translocation (*n* = 1). On the other hand, all variants identified in non-syndromic short stature are missense variants. Based on this finding, we postulate that while complete loss of the *ARID1B* gene product causes a syndrome that involves developmental delay, intellectual disability, dysmorphism and short stature, missense mutations may cause isolated short stature without developmental defects. Further testing of more non-syndromic short stature patient is warranted to validate such hypothesis.

## Conclusions

In order to evaluate the association of *ARID1B* mutations with syndromic and non-syndromic short stature, we collected data from a total of 4411 patients who underwent clinical microarray testing at Boston Children’s Hospital, 48 non-syndromic short stature patients at Shanghai Children’s Medical Center and 70 patients reported in the literature with *ARID1B* gene mutations. By case-control study, retrospective genotype-phenotype analysis and *ARID1B* gene mutation screening, we found haploinsufficient mutations of *ARID1B* are associated with syndromic short stature in Coffin-Sirissyndromeor patients with intellectual disability. *ARID1B* mutations are also found to be associated with non-syndromic short stature. This finding supports the notion that chromatin-remodeling genes play an important role in human height regulation.

### Ethics, consent and permissions

This study is reviewed and approved by ethics committee of Shanghai Children’s Medical Center【SCMC-IRB-K2013007】. All participants have signed a consent form, and all the information were obtained with appropriate consent based on the SCMC requirement.

## Additional files

Additional file 1:
**Supplementary data 1.** (DOCX 30 kb) Additional file 2:
**Supplementary data 2.** (JPEG 378 kb) Additional file 3:
**Supplementary data 3.** (DOCX 444 kb) Additional file 4:
**Supplementary data 4.** (DOCX 12 kb)

## Abbreviations

CNVs: Copy number variations
CMA: Chromosome microarray analysis
SD: Standard deviations
GWAS: Genome-wide association studies
GH: Growth hormone
MRO: Minimal region of overlap
PCR: Polymerase chain reaction
STR: Short tandem repeat
