# Supplementary material for: De novo mutations in ARID1B associated with both syndromic and non-syndromic short stature
Source: BMC Genomics. 2015 Sep 16;16(1):701. doi: 10.1186/s12864-015-1898-1 (PMC4574214; doi:10.1186/s12864-015-1898-1)
Supplement: Additional file 1: — Supplementary data 1. (DOCX 30 kb) [file 12864_2015_1898_MOESM1_ESM.docx]

**Supplemental document:**

**Clinical presentations of patients with deletion/duplication involving *ARID1B*:**

Patient A is a 10-year-old Caucasian boy who carries a 658kb*de novo* deletion at 6q25.3 (chr6:156837515 -157495889). He has a history ofglobal developmental delay andhypotonia. He receives physical therapy, occupational therapy and speech therapy and takesvalproic acid for controlling his complex partial seizure. He has good receptive language but is non-verbal. He socializes with other kids.

He is normocephalic (OFC at 50^th^ percentile). His weight is at 10^th^ percentile and his heights are consistently under 3^rd^ percentile but he is growing along his curve (Figure S1a-1).He has somewhat coarse facial featuresincludingupslanting palpebral fissures, long eyelashes, large-appearing eyes, a broad forehead, broad nasal root,anteverted nares, a short filtrum and large ear lobes.He has a large mouth, full lips and submucous cleft palate.He has very flat feet (pesplanus). MRI showed periventricular T2 signal abnormality suggesting delayed myelination and thinning of the splenium of the corpus callosum. His hip x-ray showed delayed ossification. The patient has many features of Coffin-Siris syndrome but was not noted to have hypoplastic or absent fifth fingernails or toenails.

Patient B is a 10-year-old Kuwaiti boy who carries a 6.8Mb *de novo*deletion at 6q25.1-q25.3 (Chr6: 150,653,624-157,488,885) and complex clinical phenotype. He has a history of global developmental delay, conductive hearing loss, high myopia and hypotonia. He had tethered testes for which bilateral orchiopexywas performed. He was born small (weight at 5^th^ percentile), had gross motor delay, cognitive delay and significant language delay. He receives physical therapy, occupational therapy and speech therapy. Presently he has very little language and no spontaneous speech, butengages in frequent echolalia.He had myringotomy tubes placed and a nasalseptalreconstruction.His echocardiogram showed good cardiac function but a patent foramen ovale. The electrocardiogram revealed mild intraventricular conduction delay. He was diagnosed with gastrocnemius tightness andreceived leg braces.MRI showed an abnormally smallsplenium of the corpus callosum.X-rays examination revealed scoliosis, mild curvature of the spine,prominence of T12 pediclesand symphysis diastasis.

He is microcephalic (OFC at 2^nd^ percentile) and short (height at 3^rd^ percentile, Figure S1a-2). He has somewhat course facial features including a low anterior hairline with hirsutism of the forehead, slightly prominent and fleshy ears, a depressed nasal bridge, large mouth with prominent lips. He has thick, coarse and rather kinky hair withhypertrichosis on his forehead and back. Upper extremities revealed short fingers.His lower extremities revealed valgus flat feet bilaterally and hypoplastic distal phalanges of the great toes. Neurologic examination revealed slight hypotoniabut he was alert and active.

He hasa tendency to go off and play by himself with limited social interaction.He has a fascination with mechanical objects. He enjoys playing with computers by hitting the buttons nonpurposefully. He also enjoys looking at books by turning the pages on several pages at a time. Eye contact, social referencing and joint attention are all limited. He has some repetitiveplay such as lining up playing cards on the floor. Neurodevelopmental assessment is consistent with a Pervasive Developmental Disorder, Not Otherwise Specified (DSM-IV-TR 299.8) and a Developmental Coordination Disorder (DSM-IV-TR 307.9) diagnosis.

Patient C is a 13-year-old Caucasian girl who carries a 207Kbmaternally inherited duplication at 6q25.3 (Chr6:157303164-157509622). She has a history of developmental delay but her motor and speech delay was later resolved. She has slightly low tone. She had congenital kyphoscoliosisand idiopathic short stature (normal IGF-1 and IGFBP-3 levels and provocative GH testing showed peak GH response to arginine of 12.2 ng/mL and peak response to glucagon of 16.6 ng/mL. Growth factors, cortisol, and GHBP levels are within normal limits(growth curve in Figure S1a-3). Her annualized growth velocity is 6.1 cm/year.

She is macrocephalic (OFC at 97^th^ percentile) with a prominent forehead and low-lying hairline. Her eyes are remarkable for synophrysand accommodative esotropia. Her mouth is large with an overbite.She has mild syndactyly of toes. MRI showed normal corpus callosum and an incidental finding of a 10 millimeter cystic pineal gland. There is concern that sometimes she does hand flapping if she is really excited, and there are also concerns of low attention span. She has experienced episodes of memory lapses.

**Clinical presentations of patients with sequence variants of *ARID1B*:**

Patient Dis a 4-year-old boy who carries a paternally inherited mutationc.2351C>T/p.Ser784Leu in *ARID1B* gene. His birth weight was 2550g, brderlinesmall for gestational age. His height and weight are both below -3SD (84cm and 9.5kg at the age of 3.5 years).The patient’s sister who is 122cm at the age of 8 (10-25%) also carries the variant. Both parents have height and weight within normal range. He experienced asphyxia as a newborn and has hypospadias and testicular hypoplasia. He presented with a thin layer of subcutaneous fat at 8 months. Additionally, he has half year bone age delay . The patient has a normal intelligence level. No other physical examination anomalies were detected. Provocative GH testing showed peak GH response to arginine of 15.272 ng/mL (no indication of growth hormone deficiency). Other laboratory tests including IGF-1, FT3, FT4, TSH, E2, LH and karyotyping all showed normal results.

Patient Eis a 4-year-old boy who carries a paternally inheritedmutationc.4727C>T/p.Pro1576Leu in *ARID1B* gene. His birth weight was 3800g. He has a significantly reduced growth velocity since age 3. His present height and weight are both below -2SD (97.1 cm and 17 kg at the age of 4). His parents are not short (165cm for father and 155cm for mother). The brother is 136cm at 8 years and 3 months, and did not inherit the variant. The proband has no history of chronic diseases and no physical and mental abnormalities. Provocative GH testing showed peak GH response to clonidineis5.989 ng/mL (partial growth hormone deficiency).

Patient Fisa 10-year-old girl who carries a *de novo*mutationc.4346G>C/p.Gly1449Ala in the *ARID1B* gene. Her annual growth rate is about 4 cm and she is short since early on (123.6 cm at 9 years and 8 month) but with normal weight (27.5kg at the same age). Both parents have normal heights (172 cm for father and 160cm for mother). She has a ventricular septal defect.Her intelligence is normal.Provocative GH testing showed peak GH response to arginine is5.316 ng/mL and to clonidine is 5.1, indicating partial deficiency of growth hormone. IGF-1 is normal (503.7 ng/ml).

Patient Gis a 9-year-old girl who carries a *de novo* mutation c.5998G>T/p.Asp2000Tyr in the *ARID1B* gene. Her growth velocity is approximately 5cm/year. Her height at age of 8 years and 9 months is 122.3 cm (3%), 29.5kg (50%). She has delayed bone age (equivalent to 7 years old).Both parents have normal heights (173cm for father and 159cm for mother). Provocative GH testing showed peak GH response to arginine is6.39 ng/mL, to clonidine is 3.86, indicating partial growth hormone deficiency. She has a normal physical exam and intellectual level.

**Information of 48 short stature patients recruited for sequencing**

| Subject ID | Gender | Age/year | Height/cm | Percentile | Weight/kg | Bone age by  X-ray in year |
| --- | --- | --- | --- | --- | --- | --- |
| SS-1 | Female | 5 | 99.5 | <-2SD | 15.5 | 2 |
| SS-2 | Male | 5 1/12 | 103.7 | <-2SD | 13 | 2~2 8/12 |
| SS-3 | Male | 3 2/12 | 93.7 | <-2SD | 15 | 3 2/12 |
| SS-4 | Male | 5 3/12 | 104.2 | <-2SD | 16 | 3 6/12 |
| SS-5 | Male | 4 1/12 | 95.8 | <-2SD | 15 | 2 |
| SS-6 | Female | 9 6/12 | 111 | <-2SD | 21 | 8 |
| SS-7 | Male | 4 6/12 | 95 | <-2SD | 13.4 | 3 |
| SS-8 | Male | 5 1/12 | 85.5 | <-2SD | 13.5 | 5 |
| SS-9 | Male | 8 | 100.2 | <-2SD | 14 | 2 8/12 |
| SS-10 | Male | 5 6/12 | 105.7 | <-2SD | 17 | 4+ |
| SS-11 | Female | 2 4/12 | 79.5 | <-2SD | 10.3 | 2 |
| SS-12 | Male | 4 3/12 | 89.4 | <-3SD | 11.1 | 2 |
| SS-13 | Male | 5 6/12 | 100.5 | -3SD | 15 | 4 |
| SS-14 | Male | 5 3/12 | 99.4 | -3SD | 12.5 | 4 |
| SS-15 | Female | 5 | 97.5 | -3SD | 14 | 2 6/12 |
| SS-16 | Male | 5 6/12 | 93 | -3SD | 11.5 | 2 |
| SS-17 | Male | 5 3/12 | 99.4 | -3SD | 12.5 | 4 |
| SS-18 | Male | 9 | 112.1 | <-2SD | 24 | 7 |
| SS-19 | Male | 3 8/12 | 92.3 | <-2SD | 12.1 | 2 8/12 |
| SS-20 | Male | 4 10/12 | 102.1 | -2SD | 17 | 3 6/12~4 |
| SS-21 | Female | 5 10/12 | 108.4 | -2SD | 19 | 5 9/12~6 10/12 |
| SS-22 | Male | 6 4/12 | 110.3 | <-2SD | 17.5 | 2 6/12,4 |
| SS-23 | Female | 7 3/12 | 107.4 | <-2SD | 19.3 | 7 |
| SS-24 | Male | 7 7/12 | 118.6 | <-2SD | 25.3 | 5 5/12 |
| SS-25 | Female | 7 3/12 | 107.4 | <-2SD | 19.3 | 6 |
| SS-26 | Male | 6 8/12 | 111.4 | <-2SD | 18 | 4 |
| SS-27 | Female | 4 8/12 | 98.3 | <-2SD | 13.5 | 2 6/12 |
| SS-28 | Male | 8 | 117.6 | <-2SD | 24.5 | 7 |
| SS-29 | Female | 11 | 127 | <-2SD | 20.3 | 11 |
| SS-30 | Female | 6 | 107 | <-2SD | 15.2 | 5 9/12 |
| SS-31 | Male | 6 1/12 | 105.7 | <-2SD | 17 | 5 |
| SS-32 | Female | 7 5/12 | 109 | <-2SD | 15.9 | 4 |
| SS-33 | Male | 10 11/12 | 130.5 | <-2SD | 25.5 | 9 |
| SS-34 | Male | 6 9/12 | 112.3 | <-2SD | 19.5 | 5 |
| SS-35 | Male | 9 11/12 | 123.8 | -3SD | 24 | 5-6 |
| SS-36 | Female | 11 | 131.5 | <-2SD | 24.5 | 8 10/12~10 |
| SS-37 | Female | 9 6/12 | 123.8 | <-2SD | 22.5 | 8 10/12~10 |
| SS-38 | Female | 6 6/12 | 105.5 | <-2SD | 16.3 | 5 |
| SS-39 | Male | 6 5/12 | 110.2 | <-2SD | 17 | 5-6 |
| SS-40 | Female | 8 3/12 | 120 | <-2SD | 25 | 6 10/12 |
| SS-41 | Female | 6 5/12 | 111.9 | <-2SD | 19 | 4 2/12 |
| SS-42 | Male | 6 1/12 | 106.5 | <-2SD | 18 | 3 |
| SS-43 | Male | 7 11/12 | 102.7 | <-3SD | 15 | 5 |
| SS-44 | Female | 4 8/12 | 98.4 | <-2SD | 14.5 | 3 6/12 |
| SS-45 | Male | 3 6/12 | 84 | <-3SD | 9.5 | 3 |
| SS-46 | Male | 3 11/12 | 97.1 | <-2SD | 17 | 2 8/12 |
| SS-47 | Female | 9 8/12 | 123.6 | <-2SD | 27.5 | 7 10/12 |
| SS-48 | Female | 8 9/12 | 122.3 | -1.8SD | 29.5 | 7 |
