# Supplementary figures and images for: De novo mutations in ARID1B associated with both syndromic and non-syndromic short stature
Source: BMC Genomics. 2015 Sep 16;16(1):701. doi: 10.1186/s12864-015-1898-1 (PMC4574214; doi:10.1186/s12864-015-1898-1)

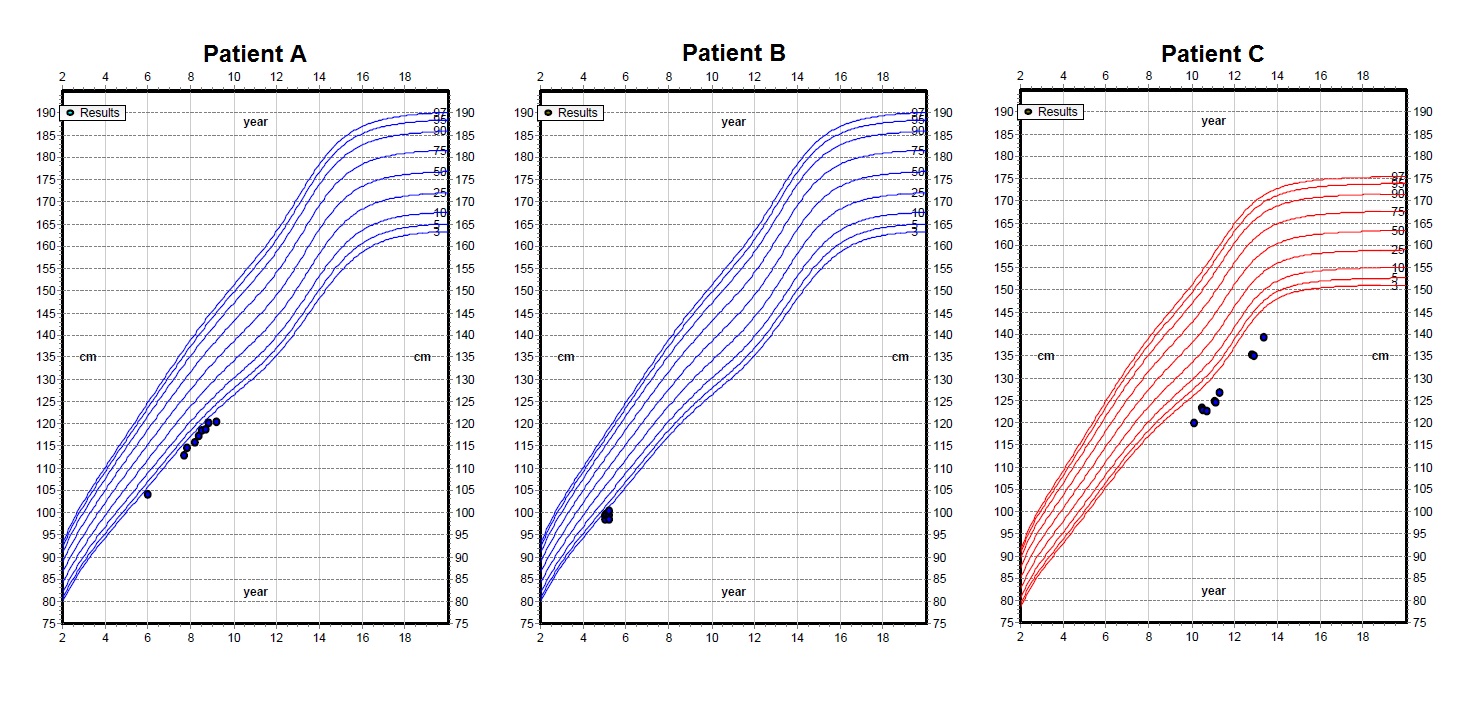

Supplement: Additional file 2: — Supplementary data 2. (JPEG 378 kb) [file 12864_2015_1898_MOESM2_ESM.jpg]
