# Supplementary material for: De novo mutations in ARID1B associated with both syndromic and non-syndromic short stature
Source: BMC Genomics. 2015 Sep 16;16(1):701. doi: 10.1186/s12864-015-1898-1 (PMC4574214; doi:10.1186/s12864-015-1898-1)

Paternity testing of the two probands with *de novo* variants

Patient F and parents: Raw STR peak results


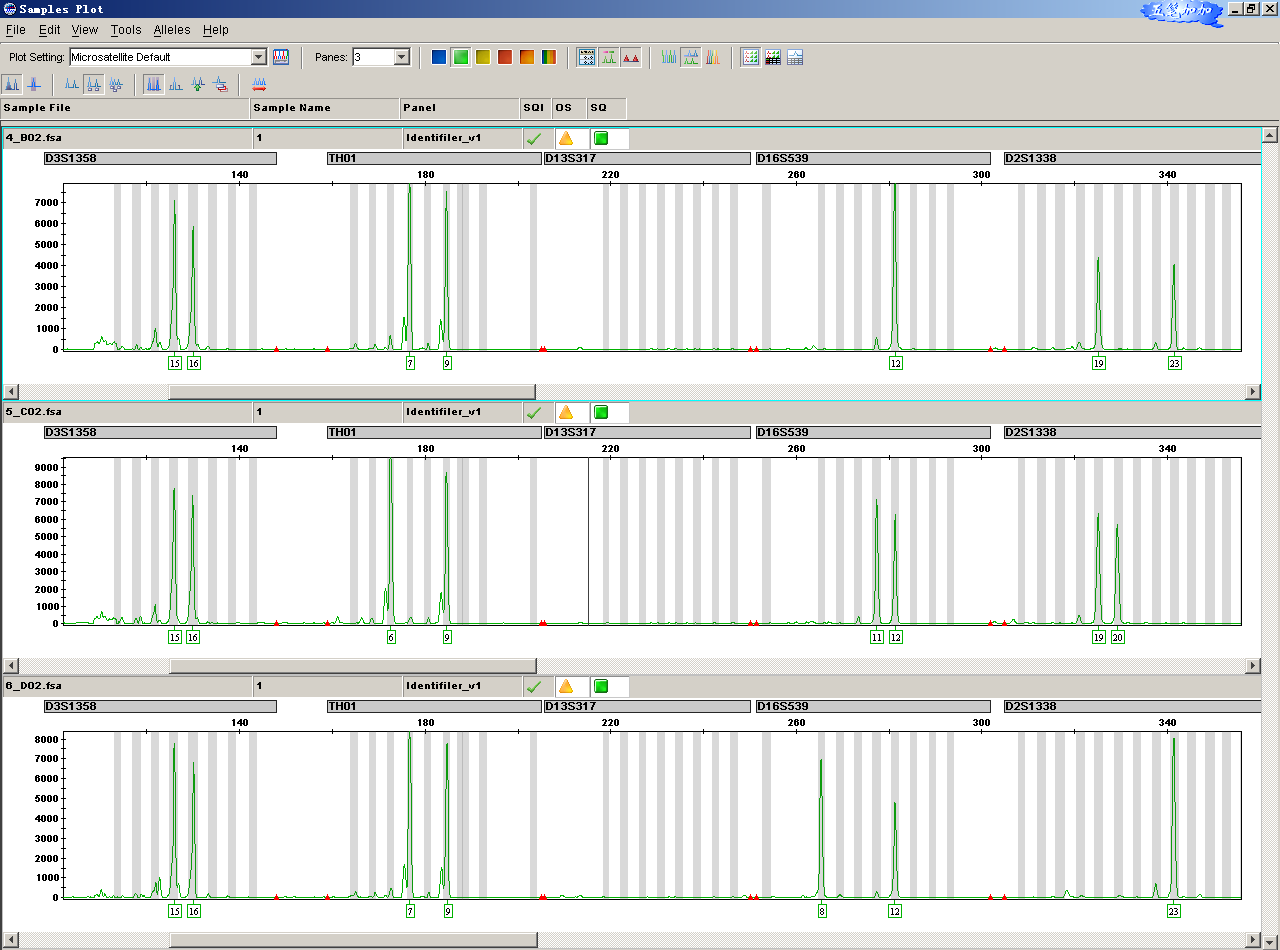


Patient F and parents: STR alleles (1.1, father; 1.2, mother; 1.3 proband)


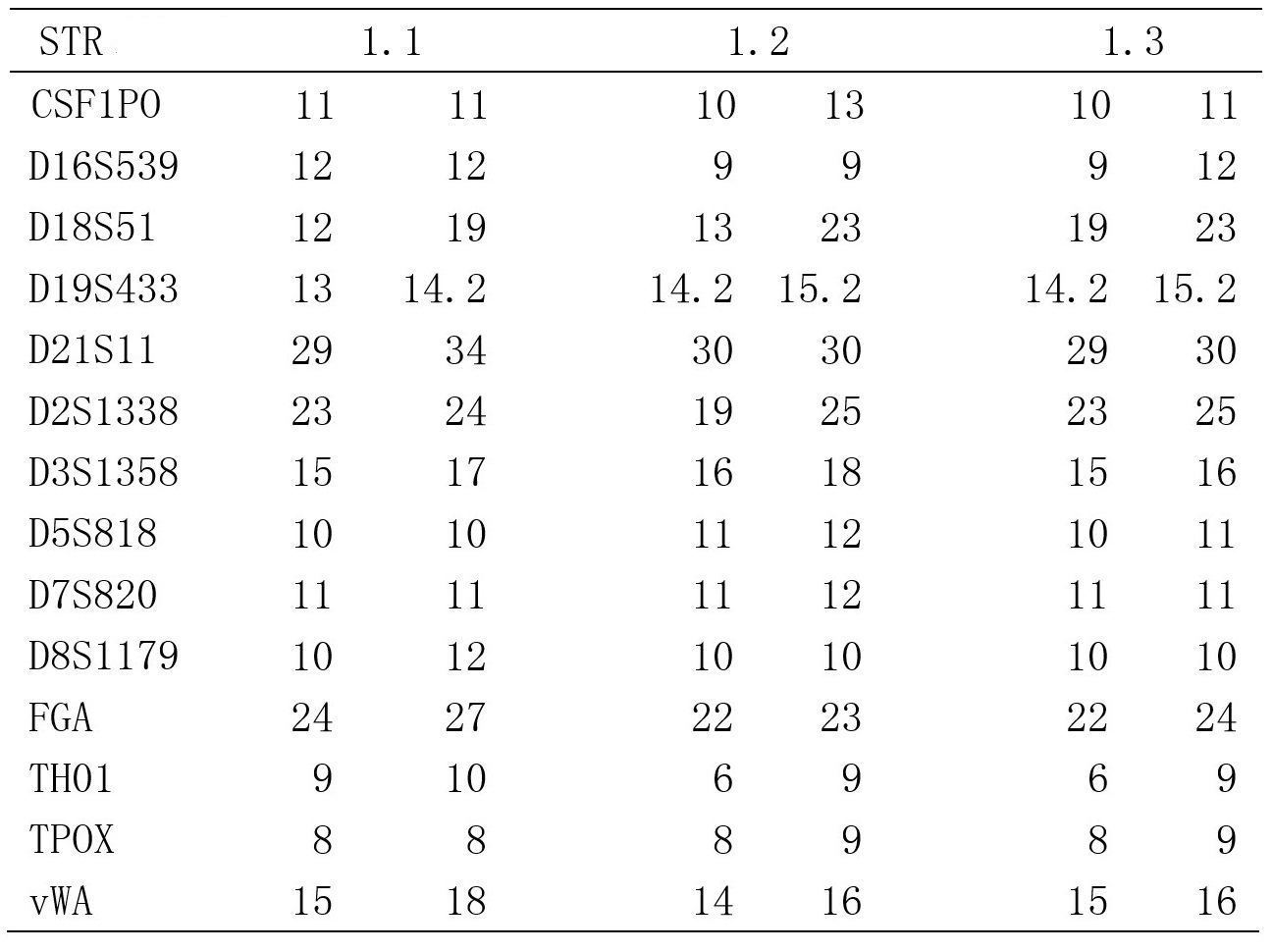


Patient G and parents:: Raw STR peak results


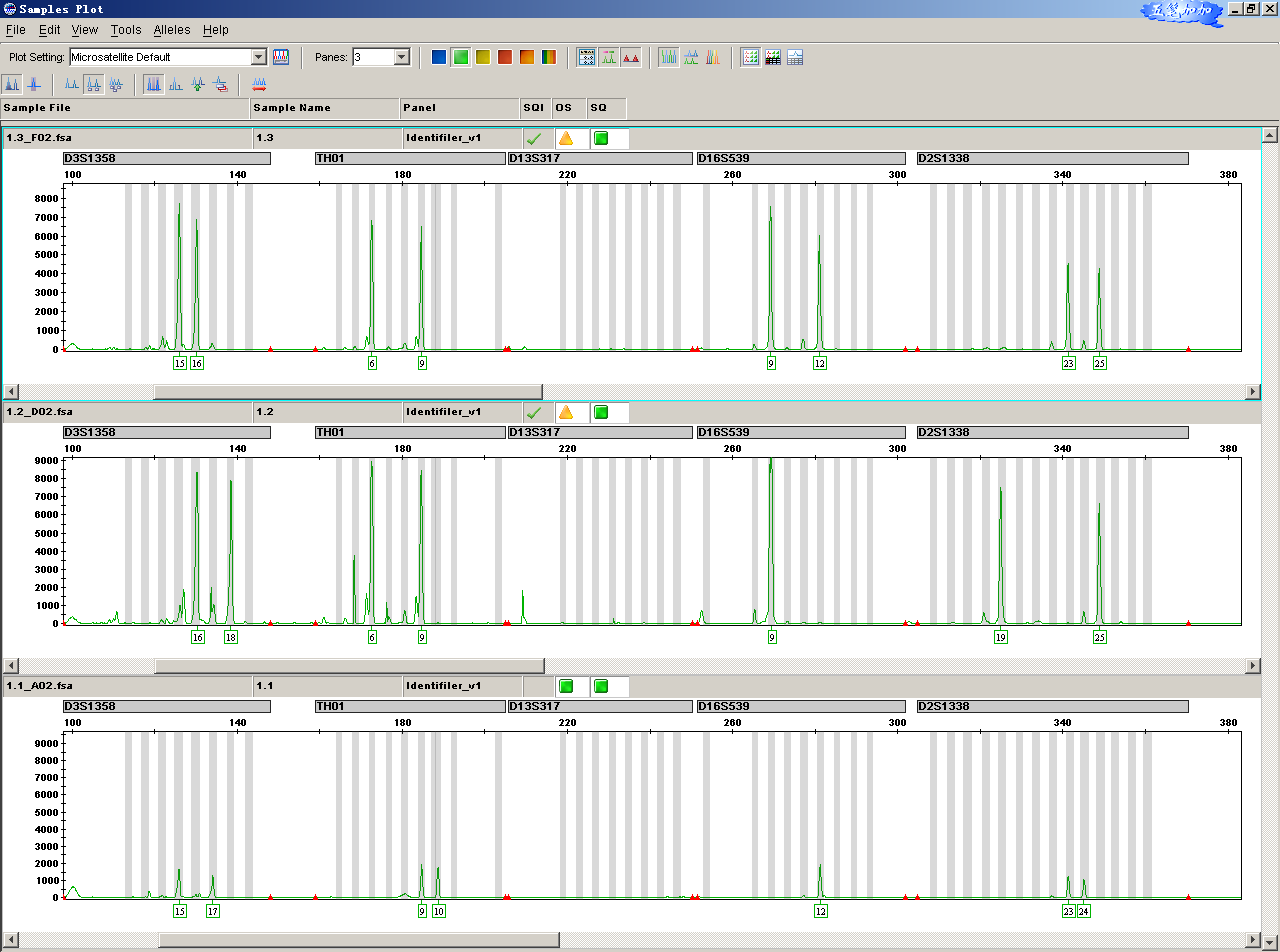


Patient G and parents: STR alleles (2.4, proband; 2.5, father; 2.6, mother)


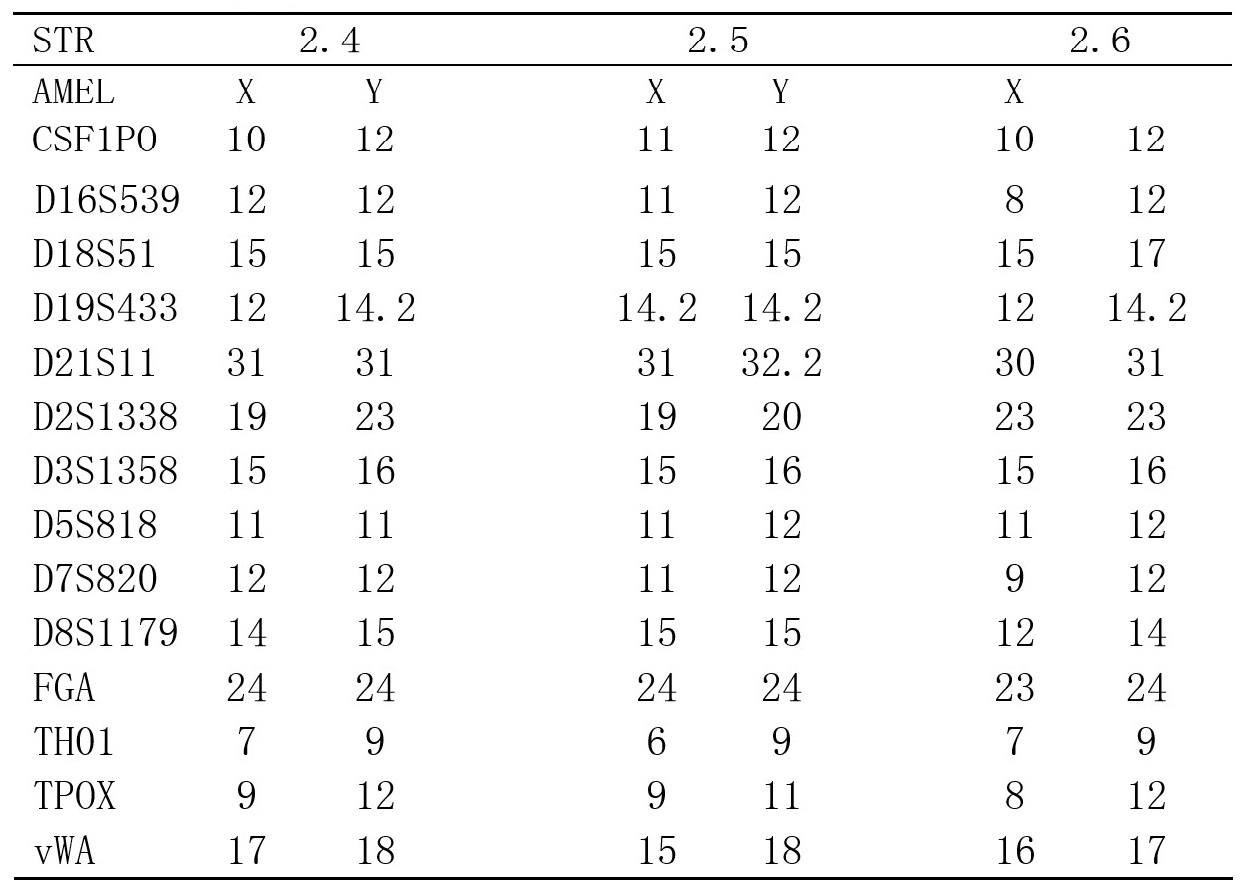

Supplement: Additional file 3: — Supplementary data 3. (DOCX 444 kb) [file 12864_2015_1898_MOESM3_ESM.docx]
