# Supplementary material for: De novo mutations in ARID1B associated with both syndromic and non-syndromic short stature
Source: BMC Genomics. 2015 Sep 16;16(1):701. doi: 10.1186/s12864-015-1898-1 (PMC4574214; doi:10.1186/s12864-015-1898-1)
Supplement: Additional file 4: — Supplementary data 4. (DOCX 12 kb) [file 12864_2015_1898_MOESM4_ESM.docx]

| Variant | origin | Nucleotide conservation | Amino acid conservation | Physiological difference between amino acids | GVGD | Polyphen 2 | SIFT | CONDEL | ACMG classification |
| --- | --- | --- | --- | --- | --- | --- | --- | --- | --- |
| c.2351C>T/p.S784L | paternal | Moderate (Polylop:4.16) | High conserved | Large (Grantham dist:145[0-215]) | Class C0 (GV:208.55-GD:106.06) | Deleterious (0.904) | Deleterious (score: 0.00) | Deleterious (0.955) | Uncertain (PM2+PP3) |
| c.4346G>C/p.G1449A | De novo | Moderate (Polylop:3.68) | Highly conserved | Small (Grantham dist:60) | Class C0 (GV:206.04-GD:31.89) | Deleterious (0.979) | Deleterious (score: 0.00) | Deleterious (0.979) | Likely pathogenic (PS2+PM2+PP3) |
| c.4727C>T/p.P1576L | paternal | Moderate (polylop:2.47) | Highly conserved | Moderate (Grantham dist:98) | Class C0 (GV: 208.63 - GD: 94.04) | Deleterious (0.975) | Deleterious (score: 0.00) | Deleterious (0.975) | Uncertain  (PM2+PP3) |
| c.5998G>T/p.D2000Y | De novo | High (polylop:4.32) | Moderate | Large (Grantham dist:160) | Class C0 (GV: 226.93 - GD: 86.68) | Deleterious (0.952) | Deleterious (score: 0.00) | Deleterious (0.952) | Likely pathogenic (PS2+PM2+PP3) |
